# Impaired hippocampal-cortical coupling but preserved local synchrony during sleep in APP/PS1 mice modeling Alzheimer’s disease

**DOI:** 10.1038/s41598-019-41851-5

**Published:** 2019-03-29

**Authors:** E. Zhurakovskaya, I. Ishchenko, I. Gureviciene, R. Aliev, O. Gröhn, H. Tanila

**Affiliations:** 1 A. I. Virtanen Institute, University of Eastern Finland, Kuopio, Finland; 2 D.I. Ivanovsky Academy of Biology and Biotechnology, Southern Federal University, Rostov-on-Don, Russian Federation; 3 Moscow Institute of Physics and Technology, Moscow, Russian Federation; 4 Institute of Theoretical and Experimental Biophysics, Puschino, Russia

## Abstract

Sleep, in addition to its brain restorative processes, plays an important role in memory transfer from its temporary store in the hippocampus to the more permanent storage in the neocortex. Alzheimer’s disease (AD) affects memory and sleep. The aim of this study was to explore disturbances in global and local synchrony patterns between brain regions in the APP/PS1 mouse model of the AD during natural sleep. We used 8 male APPswe/PS1dE9 mice and 6 wild-type littermates, aged 5–6 months, with multiple electrode bundles implanted into cortical regions, thalamus and hippocampus. We measured video-EEG in freely moving animals and analyzed synchrony during NREM vs REM sleep. Global synchrony between medial frontal cortex and hippocampus measured with magnitude-squared coherence was slightly decreased in delta range during NREM stage of sleep in APP/PS1 mice. In contrast, local hippocampal synchrony measured with cross-frequency coupling remained intact. Ripple structure or frequency did not differ between the genotypes. However, the coupling of the spindle-band power peak in the medial prefrontal cortex to hippocampal ripples was significantly decreased compared to wild-type animals. The delicate timing of hippocampal ripples, frontal delta, and corticothalamic spindle oscillations may be the first sign of impaired memory in amyloid plaque-forming transgenic mice.

## Introduction

Amyloid deposition in the brain in early stages of Alzheimer’s disease (AD) seems to concentrate on a network of highly interconnected cortical hubs that has been named default mode network (DMN) due to its high activity when the person is not interacting with the outside world and deactivation during cognitive processing^1^. The DMN regions, medial frontal, posterior cingulate and lateral parietal cortices are strongly connected to the medial temporal lobe memory system, including the hippocampus^2,3^. Assessment of functional connectivity with BOLD-fMRI has demonstrated abnormal activity in the DMN in AD patients^4^ and subjects at high risk of developing AD^5,6^. Such changes may reflect early synaptic changes in neural networks. However, except in the rare cases of carriers of causative AD mutations, it is practically impossible to get access to the earliest synaptic and network changes in the AD pathology in human subjects with currently available techniques. Here, animal models are indispensable.

Recently, several attempts have been made to visualize functional connectivity during the resting state in transgenic AD model mice using BOLD-fMRI. Differences in functional connectivity have been reported between transgenic mice and their wild-type controls, but the findings appear controversial including hypersynchrony^7,8^, no change^9^, or decreased functional connectivity^9^ between non-adjacent brain regions. However, a major obstacle in comparing these findings with human imaging studies is the use of anesthesia in the animal studies as the’resting state’. Both we and others have demonstrated that certain anesthesia induces a state in rodents that closely resembles natural sleep with identified REM and NREM epochs^10,11^. Therefore, sleep may provide the brain state that would best allow translational studies on brain functional connectivity across species.

Another reason to focus on sleep in studying the pathophysiology of AD is the proposed key role of sleep in memory consolidation at the systems level. Studies in amnesia patients have demonstrated that the hippocampus serves only as a temporary storage for memory while the long-term memory traces are assumed to reside in cortical networks. During slow-wave sleep in rodents, the prominent hippocampal sharp-wave ripple (SWR) oscillation and cortical delta (1–4 Hz) and spindle (12–18 Hz) oscillations show a correlation in a fine time scale^12,13^. Simultaneous unit firing in the hippocampus and cortical areas and correlation of the firing order of location-specific unit in these structures have led to the notion that these epochs of slow-wave sleep provide the windows for transfer of memories between the hippocampus and cortex^14^. Since sleep pattern is perturbed in an early stage of AD^15^, it is plausible that impaired information transfer between the hippocampus and cortex during sleep contribute to the most characteristic symptom of the early AD, the inability to form long-lasting memories.

The present study set out to explore differences in global and local synchrony and communication between brain regions during sleep in the widely used APPswe/PS1dE9 mouse model of AD. In this strain, amyloid-β (Aβ) deposition starts around at 4 months of age^16^ when the mice also show EEG hyperactivity^17^ and occasional epileptic seizures^18^. As in the human AD, however, memory impairment becomes manifest in behavioral studies in these mice only after the amyloid pathology has fully developed, around 10–12 months of age^19^. We employed here a new ‘stereo-EEG’ approach, double- or triple-wire electrodes distributed across key brain sites corresponding the human DMN to assess both local and interregional synchrony of extracellular oscillations.

## Results

The study was conducted in 8 male APPswe/PS1dE9 mice and 6 wild-type littermates. The age of each animal was 5–6 months. To explore connectivity between the brain regions, we inserted wire electrodes into the hippocampus, cortex, and thalamus (Suppl. Fig. 1). Histology revealed that most of the electrodes hit the intended location, with an exception of CA3 electrodes that ended up being more medially located, targeting mainly CA1 pyramidal cell layer and dentate hilus. The final location of the electrodes in each mouse are summarized in Suppl. Fig. 1. At this age, the mice showed consistent amyloid plaque deposition in the cortex and hippocampus, and some small plaques also in the dorsal thalamus (Suppl. Fig. 2).

### Increased power spectral density in TG mice

**Figure 1:**
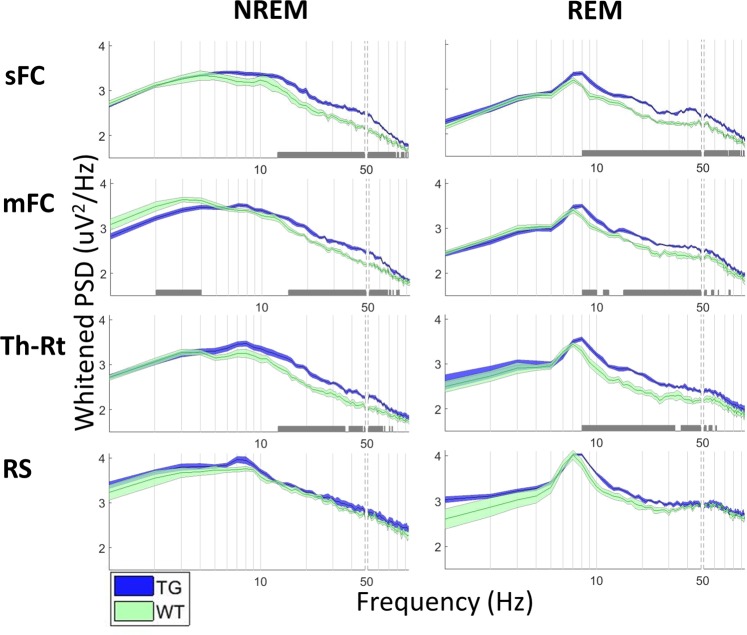
Power spectral density with standard error mean for TG (blue) and WT (green) mice during NREM and REM. MC – motor cortex, mFC – medial frontal cortex, Th-RT – thalamic reticular nucleus, RS – retrosplenial cortex. Power spectral density was whitened by multiplying with frequency for better visualization. The vertical dashed lines denote removed line frequency (50 Hz). Statistically significant differences between the genotypes are denoted with a grey bar above the x-axis (p < 0.05, FDR-corrected for multiple comparisons). The number of animals in each test: sFC: 8 TG and 5 WT animals, mFC: 8 TG and 5 WT animals, Th-RT: 7 TG and 5 WT animals, RS: 6 TG and 4 WT animals.

We estimated power spectrum for REM and NREM sleep states. Transgenic mice showed higher power spectral density than wild-type (WT) mice between 15 and ~70 Hz in cortex and thalamus during NREM sleep (Fig. 1). During REM sleep, the frontal screw electrode showed an increase in TG mice in all frequencies above the theta peak (7 Hz), while the significant increase in medial frontal cortex and thalamic reticular nucleus was limited to a band between 15 and 50 Hz (Fig. 1). These results are consistent with previous reports from the same APPswe/PS1dE9 mouse line^17^. There were no significant differences between the genotypes in the spectra of other brain regions.

### Decreased long-range connectivity between the hippocampus and frontal cortex during NREM sleep in TG mice but preserved intrahippocampal connectivity

**Figure 2:**
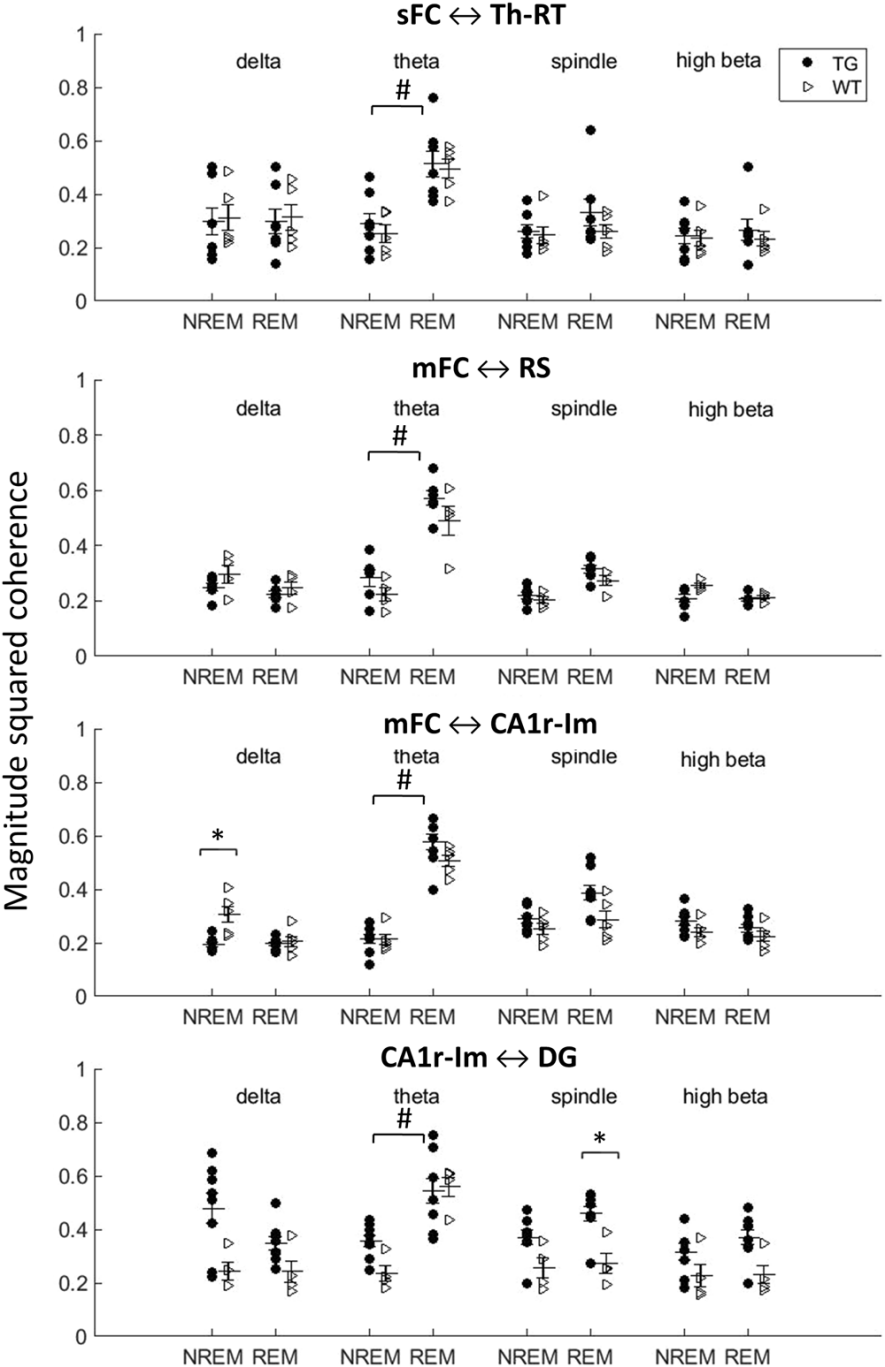
Connectivity between brain regions measured with magnitude squared coherence during NREM and REM stages of natural sleep in delta, theta, spindle and high-beta frequency bands. Connectivity between medial frontal cortex and hippocampal CA1 during NREM sleep was significantly decreased in TG mice in the delta range compared to WT mice. On the other hand, connectivity between hippocampal CA1 and DG areas was significantly increased in the spindle range in TG animals. (*p < 0.05, FDR-corrected for multiple comparisons). In addition, there was a significant connectivity increase in the theta band from NREM to REM in all three pairs of brain regions (^#^p < 0.05, FDR-corrected for multiple comparisons). The number of animals used (from top to bottom picture): 6 TG and 4 WT, 6 TG and 4 WT, 8 TG and 5 WT, 8 TG and 4 WT.

Next, we assessed long-range connectivity using magnitude-squared coherence in the default mode network (DMN) in the following frequency bands: delta (1–4 Hz), theta (5–9 Hz), spindle (10–18 Hz) and high beta (19–30 Hz). As expected, there was a significant increase in connectivity in the theta band from NREM to REM state in all pairs of brain regions examined in both TG and WT mice (Fig. 2). Mostly, the interregional connectivity in the DMN in TG mice did not differ from WT mice. However, connectivity in the delta band between mFC and CA1 was significantly lower in TG than in WT mice during NREM sleep (Fig. 2). Since a previous study^20^ also found impaired long-range coherence in another APP/PS1 line during anesthesia, we further examined the mFC – CA1 connectivity in the delta band at three concentrations of isoflurane (1–1.6%). A similar trend toward decreased delta-range connectivity in TG mice was observed, but it did not reach significance during anesthesia (Suppl. Fig. 3). Further, we assessed intrahippocampal connectivity, more precisely between hippocampal CA1 and dentate gyrus (DG) subregions. During NREM, the majority of TG mice displayed a higher connectivity than WT mice in the delta, theta, and spindle bands, but the remaining TG mice had values within the range of WT mice (Fig. 2). During REM, all but one TG mice showed a higher connectivity in alpha and beta bands than WT mice (Fig. 2), but due to dispersion within the TG mouse group, the difference did not reach significance.

### Cross-frequency coupling patterns are not disturbed in TG animals

**Figure 3:**
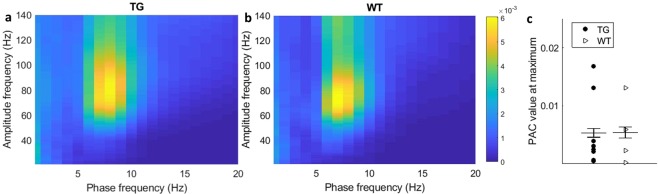
Phase-amplitude coupling during REM state in the dentate gyrus in TG (**a**) and WT (**b**) mice. (**c**) The mean value of PAC in the region of high coupling (phase frequency 7–8 Hz, amplitude frequency 60–90 Hz) did not differ between the genotypes (p = 0.98, two-sample t-test). The number of animals used: 8 TG and 4 WT.

Cross-frequency coupling is suggested to play an important role in the information transfer process^21^. Possible disturbances in the coupling pattern might be a sign of memory problems. Strong cross-frequency coupling, i.e. between the theta phase and gamma amplitude, was present in the hippocampus during REM state (Fig. 3). However, the coupling increase did not differ between the genotypes.

### Normal CA1 ripple structure and frequency but attenuated hippocampal ripple – cortical spindle coupling of TG mice during NREM sleep

**Figure 4:**
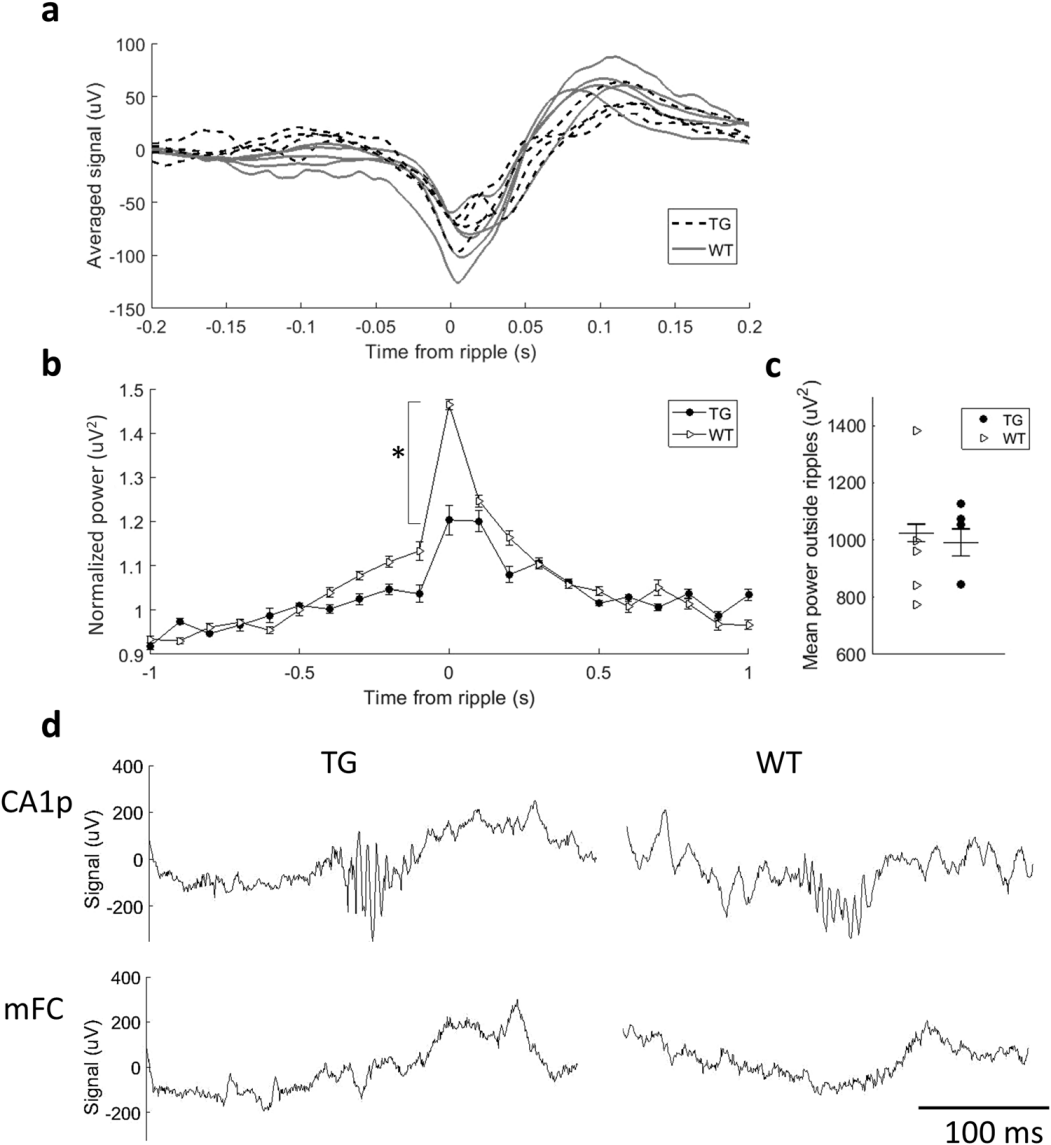
(**a**) Average low frequency (1–50 Hz) cortical response in the medial frontal cortex triggered from the peak of the hippocampal ripple. (**b**) Averaged normalized power in the spindle band (10–18 Hz) in the medial frontal cortex around the hippocampal ripple with standard error mean. The power was normalized in relation to the mean power in the same animals during 2 s around the midpoint between each pair of the hippocampal ripples. The peak power around the ripple was statistically higher in WT animals (p = 0.01, two-sample t-test). (**c**) Averaged mean power outside ripples for individual animals. There was no difference between the genotypes (p = 0.8). The number of animals for (**a**), (**b**) and (**c**) comparisons: 4 TG and 5 WT. The number of ripples (**b**) and intervals between ripples (**c**) used for averaging: 585. (**d**) Representative examples of a ripple event in the CA1 pyramidal cell layer (top) and a corresponding trace from the frontal cortex from TG and WT animals (bottom).

Functional coupling between the hippocampus and neocortex during NREM sleep is considered a key mechanism for systems-level memory consolidation^14^. An essential factor in this memory transfer from the hippocampus to neocortex is thought to be the functional coupling between sharp-wave ripples on CA1 and delta- and spindle oscillations in the cortex^12,22^. In 5 WT and 4 TG mice, the CA1 electrodes hit the pyramidal cell layer so that 150–250 Hz ripples were reliably detected, allowing us to compare the ripples and their functional coupling with the medial frontal cortex. The ripple duration, intraripple frequency, and ripple occurrence did not differ between the genotypes (Table 1). Using the ripple-peak as a trigger, we averaged cortical (mFC) low-frequency response (1–50 Hz) from 765 ripples in each mouse. The mean response dominated by the cortical down-state, or intracortical positivity^12^ peaking at ~100 ms after the ripple peak did not differ between the genotypes (Fig. 4a). However, the ripple-centered spindle power was significantly attenuated in TG mice compared to WT mice (Fig. 4b), whereas the spindle power between the ripples did not differ between the genotypes (Fig. 4c).

**Table 1:** Properties of CA1 ripple oscillations during NREM sleep and waking immobility (WI) in APP/PS1 transgenic and wild-type mice. Data are given as mean ± SEM.

	NREM		WI	
	TG	WT	TG	WT
Ripples/min	8.2 ± 6.7	9.3 ± 6.9	3.7 ± 2.9	3.7 ± 2.9
Ripple frequency, Hz	150.7 ± 1.7	152.1 ± 3.3	153.4 ± 4.5	154.4 ± 3.3
Ripple duration, ms	47.2 ± 6	48.5 ± 4.2	46.5 ± 7.4	49.1 ± 3.7

## Discussion

This study addressed long-range functional connectivity during sleep in brains of transgenic APP/PS1 mice modeling Alzheimer’s disease (AD) and their wild-type littermates using a novel electrophysiological approach, implantation of distributed intracerebral doublet or triples wire electrodes in the regions of interest. We focused on the connectivity of the frontal cortex with the hippocampus, thalamus, and retrosplenial cortex, the latter being the key hub for the default mode network in humans. Whereas local connectivity in the hippocampus was well preserved in APP/PS1 mice, impaired hippocampal – medial frontal cortical coupling was evident in APP/PS1 mice at an age (5–6 months) when amyloid pathology is present in the cortex and hippocampus^16^ but memory impairment has not become manifest in behavioral tasks^19^.

Consistent with our previous study in slightly younger (4–5 months) APP/PS1 mice^17^, we observed increased power spectral density from between 10–60 Hz in both NREM and REM states in frontal cortex and thalamus, and marginally in the hippocampal CA1 but not in the DG subregion. A new finding was no increase in power spectral density in the retrosplenial cortex. These findings suggest that cortical hyperexcitability in this mouse model is frontally biased, despite similar amyloid load in the frontal and posterior cortical regions^16^.

A recent BOLD-fMRI follow-up study on APPswe (Tg2576) transgenic mice demonstrated increased functional coupling of the hippocampal network (hippocampus as a seed) as well as other components of the default-mode network at the age of 5 months, when these mice do not yet display amyloid plaques, but decreased functional coupling in the hippocampal network starting from 8 to 18 months of age when amyloid plaques are present^7^. This finding is largely consistent with another BOLD-fMRI study on the same APPswe/PS1dE9 mouse as ours, showing reduced local functional coupling in the somatomotor cortex of 15-month-old TG mice compared to wild-type controls^9^. In both studies, the mice were slightly sedated (medetomidine +0.5% isoflurane). The idea of impaired long-range functional connectivity in amyloid plaque bearing transgenic mice is further supported by an *in vivo* calcium imaging study employing another APP/PS1 model (APP23 × PS45) with a similar time course of amyloid accumulation as the APPswe/PS1dE9 mouse. This study reported impaired coherence of slow-wave oscillations between cortical regions as well as between the frontal cortex and the hippocampus in 6- to 8-month-old APP/PS1 mice under light isoflurane anesthesia (0.8–1%)^23^. Whereas wild-type mice showed the high coherence of slow-waves across the entire cortex, the coherence dramatically decreased as a function of the distance between recording sites in the APP/PS1 mice. Unfortunately, the conclusion was somewhat undermined by the fact that the frontal cortex that was the reference for all long-range coherence measures, showed abnormally high and irregular local oscillation in contrast to regular up-state, down-state pattern of the wild-type mouse frontal cortex^23^.

Our finding of preserved intrahippocampal connectivity across all ranges of significant oscillation but impaired slow-wave connectivity (delta-band) between hippocampal CA1 and medial frontal cortex during NREM sleep in APP/PS1 mice is consistent with the main finding of impaired long-range functional connectivity of the above-mentioned imaging studies. Although the impaired connectivity was no longer statistically significant in our additional experiments under isoflurane anesthesia, the trend was the same. Further, Busche *et al*.^20^ demonstrated that the impaired long-range coherence in APP/PS1 mice could be similarly observed under light isoflurane anesthesia and natural NREM sleep. However, our finding of unimpaired connectivity between two other long-range nodes, the medial frontal and retrosplenial cortices, suggests that, at least in the early state of amyloid pathology, the perturbation of functional coupling is not a simple function of pathway length but more selective. Despite decreased connectivity between CA1 and medial frontal cortex over the delta frequency band, our analysis of ripple-triggered mean responses in the medial frontal cortex showed similar intracortical positive (functional down-state) delta wave in APP/PS1 mice as in their wild-type littermates. However, the associated increase in cortical spindle power (10–18 Hz) was significantly attenuated in APP/PS1 mice.

It has long been speculated that the alignment of neocortical (especially prefrontal) delta oscillation, hippocampal sharp-wave ripple activity, and cortico-thalamic spindle oscillation constitute the critical functional coupling that allows transfer of temporarily stored memories from the hippocampus to long-term storage in the cortex^12,14^. Indeed, a recent study demonstrated that optogenetically induced spindles during NREM sleep in mice enhance one-trial memory consolidation (contextual fear conditioning, assessed 24 h after learning) when given in phase with the cortical up-state (surface-negative delta-wave) but not when given out of phase. Vice versa, optogenetic perturbation of sleep spindles in phase with the cortical up state, but not when given out of phase, impaired memory consolidation^22^. It is well possible that the impaired coupling of frontal cortical sleep spindles with hippocampal ripples found in the present study represents the first mechanism leading to memory impairment in AD model mice and human with mild-cognitive impairment. Interestingly, the first memory impairment observed in APP transgenic mice is in their retention of spatial memory overnight^24,25^. Moreover, a very recent study reported impaired phase coupling of sleep spindle with delta-oscillation in older adults compared to young ones. Moreover, the time-shift in the phase coupling correlated with the ability to remember nonsense word-pairs overnight, and further correlated with the decreased volume of the medial frontal cortex^26^.

To conclude, it appears that the ability to strengthen memories during NREM sleep depends on the functional coupling between frontal delta-oscillations, hippocampal ripple oscillation, and thalamocortical spindle oscillations. These delicate clockworks depending on two long-range neural circuits may be the most sensitive type of memory mechanism for age-associated degenerative changes in the brain, including Alzheimer-like brain amyloidosis. With the advent of functional MRI and multielectrode *in vivo* electrophysiology applicable in mice in combination with the present huge toolbox for genetic manipulations, it is likely that our understanding of these clockworks will dramatically increase in coming years and lead to new treatment options for age-related memory impairment.

## Materials and Methods

### Animals

The subjects were 8 male transgenic (TG) APPswe/PS1dE9 mice^27^ and 6 wild-type (WT) littermates. The weight of each animal was 27–30 g, age 5–6 months. The APP/PS1 colony was based on founders obtained from D. Borchelt and J. Jankowsky (Johns Hopkins University, Baltimore, MD, USA). The mice were backcrossed to C57BL/6 J strain for 18 generations. After electrode implantation, the animals were kept in single standard laboratory cages in a controlled environment (constant temperature, 22 ± 1 °C, humidity 50–60%, lights on 07:00–19:00), with food and water available ad libitum. All animal procedures were carried out in accordance with the guidelines of the European Community Council Directives 86/609/EEC and approved by the Animal Experiment Board of Finland.

### Electrode implantation

For long-term EEG monitoring mice were implanted with 15 electrodes into the different brain areas (Suppl. Fig. 1). Two screw electrodes (diameter 1.0 mm, length 2.0 mm, Microbiotech/se AB) were fixed bilaterally on the frontal bone at AP 2.7 mm, ML ± 2 mm from bregma. Two parietal screw electrodes were implanted bilaterally on the occipital bone above the cerebellum and were used as ground and reference electrodes. The screws served also as anchors for dental acrylic cement and the connector (Mill-Max, NY, USA). For recording deep brain regions, we implanted wire electrodes (Formwar insulated stainless steel wire, diameter 50 µm, California Fine Wire Company Co, Grover Beach, CA, USA). A pair of wire electrodes was aimed at the medial frontal cortex (AP 1.4, ML 0.4 and 0.9 mm from bregma). Double electrodes with a vertical tip separation of 400 µm were aimed at reticular thalamic nucleus (Th-Rt, AP −1.1, ML − 1.7), CA3 layer of the hippocampus (AP 2.1, ML + 2.4), and retrosplenial cortex (RSC, AP −2.9 mm, ML + 0.5). A triple wire electrode with a vertical tip separation of 400 µm was implanted in the CA1 layer (AP −2.1 mm, ML − 1.5). In addition, a silver wire (perfluoroalkoxy-insulated, diameter 200 µm, A-M Systems, Sequim, WA, USA) was inserted into the neck muscles during surgery for electromyogram (EMG) recording.

The operation was done under general isoflurane anesthesia (induction 4.5%, maintenance at 1.8–2.1%). After the surgery, the mice received carprofen (5 mg/kg, s.c., Rimadyl®, Vericore, Dundee, UK) for postoperative analgesia, and antibiotic powder (bacitrasin 250 IU/g and neomycinsulfate 5 mg/g, Bacibact, Orion, Finland) was applied. Mice were allowed to recover for 10 ± 3 days before the recording started.

### Recording protocol

EEG-video recordings were conducted on freely moving animals during the light period in 3-h sessions. During the recordings, the mice were in standard plastic cages (width 18 cm × length 21 cm × height 30 cm) and connected to an 18-ch headstage preamplifier with a light-weighted recording cable (Plexon Inc., Dallas, TX, USA). The signal was further amplified with an AC amplifier (gain 1000, bandpass-filtering 1–3000 Hz, Grass, Quincy, MA, USA). The signal was digitized at 2 kHz per channel (DT2821 series A/D board; Data Translation, Marlboro, MA, USA) and acquired by Sciworks 5.0 program (DataWave Technologies, Loveland, CO, USA). The behavior of the animals was video recorded with an overhead video camera (Live!Cam, Video IM Pro, Creative, Dublin, Ireland) synchronized with electrophysiological signals. Each mouse underwent 4 sessions of 3-h recordings.

In addition, in order to estimate connectivity under anesthesia, each mouse underwent recording under isoflurane using the following scheme: 5 minutes of 2% isoflurane, 10 minutes of 1.6% isoflurane, 10 minutes of 1.3% isoflurane, 10 minutes of 1% isoflurane, 10 minutes of 1.6% isoflurane, 10 minutes of 1.3% isoflurane, 10 minutes of 2% isoflurane and 10 minutes of 1% isoflurane.

### Data analysis

All data analysis was performed using custom scripts written in Matlab R2017b (Mathworks, Natick, MA, USA). For each mouse, the sleep states were detected automatically based on EEG and EMG recordings similarly to^28^. For sleep states detection, recordings were divided into 2 s bins, and then the frequency content of the cortical screw channel was estimated using the short-time Fourier transform for each bin. The following rules were applied to each bin to classify it as NREM, REM, waking immobility (WI) or movement state. If the maximum EMG value was higher than the predefined threshold (2 × SD), the bin was classified as a movement. The threshold for EMG was additionally confirmed and adjusted with video recordings for each animal. If alpha to gamma power ratio was higher than the mean value, the bin was classified as NREM. If theta to delta power ratio was higher than twice the mean value, the bin was classified as REM. Neighboring REM bins were combined if theta to delta ratio between them was still higher than the mean value, and the animal was not moving. Two NREM bins were merged together if alpha to gamma ratio between them was not below half of the mean value, and the animal was not moving. All other bins were classified as WI. For the connectivity analysis purposes, we were interested only in NREM and REM epochs. We took 20 s (i.e. 10 consecutive bins) of each REM state and 20 s of preceding NREM state, with the exclusion of 20 s of the transitional pre-REM epoch^29^.

*Power spectra* were calculated for each 20 s REM and NREM epoch using the Welch’s power spectral density estimate and averaged for each animal.

*Synchronization* was estimated for corticohippocampal, intrahippocampal and intracortical connections using magnitude-squared coherence^30^. This value shows how well signals correspond at each frequency. It takes values between 0 and 1 and is calculated from cross spectrum P_xy_(f) normalized by power spectra P_xx_(f) and P_yy_(f).

$${C}_{xy(f)}=\frac{|{P}_{xy}(f)|}{{P}_{xx}(f){P}_{yy}(f)}$$

Four frequency bands were chosen to estimate synchronization: delta (1–4 Hz), theta (5–9 Hz), spindle (10–18 Hz) and high beta (19–30 Hz). The magnitude squared coherence was averaged in these intervals.

In addition, synchronization was calculated under isoflurane. From each 10-min step of isoflurane concentration, we analyzed last 5 min to allow anesthesia effect to stabilize. Magnitude-squared coherence was calculated and averaged for each isoflurane concentration. Data for 2% concentration is not shown, as coherence is highly affected by the typical burst-suppression pattern.

One of the methods to study brain activity is *phase-amplitude coupling*. It detects situations when low-frequency signal phase changes simultaneously with high-frequency signal power. We calculated phase-amplitude coupling during REM stage in the hippocampus and compared it between the genotypes using the PAC toolbox based on modulation index calculation^31^.

*Sharp-wave ripples* (SWRs) were detected automatically using FMAToolbox (http://fmatoolbox.sourceforge.net/) based on power in the 150–250 Hz band and duration (50–100 ms). For oscillation to be detected as a ripple, it needed to have an amplitude from 2 to 5 SD above the baseline. After detection, the ripples were checked manually to distinguish them from spiking activity. Ripples occurred during WI and NREM epochs. Mean ripple duration, intraripple frequency, and ripple occurrence rate were calculated for all animals.

In the analysis of frontal cortical responses to hippocampal SWRs, we took only ripples that happened during NREM state. To have the same number of ripples for each mouse, we limited the number of ripples to 585. Ripple-triggered average activity in the cortex was calculated for spindle (10–18 Hz) power and compared between the genotypes. Power around ripples was calculated and averaged for each animal. Power was normalized using averaged power from 585 2s-long epochs between ripples (epochs around the mid-point between ripples).

### Experimental Design and Statistical Analysis

The basic design of the experiment was to compare male APP/PS1 transgenic mice to their wild-type littermates at the same age. The person carrying out the data collection was blind to the animal genotype. Only data from electrodes that were histologically and electrophysiologically confirmed to have the same location were included in the comparison, which resulted in an unequal number of cases in various statistical comparisons.

Power spectra group comparisons were performed using a two-sample t-test with false discovery rate (FDR) correction for multiple comparisons^32^. Statistical tests for magnitude-squared coherence were performed using permutation tests with FDR correction for multiple comparisons. Ripple parameters and phase-amplitude coupling values were compared between transgenic and wild-type groups using two-sample t-test.

## Supplementary information

Supplementary figures and legends clean

## Data Availability

The datasets generated and analyzed during the current study are available from the corresponding author on reasonable request.
