# Supplementary material for: Impaired hippocampal-cortical coupling but preserved local synchrony during sleep in APP/PS1 mice modeling Alzheimer’s disease
Source: Sci Rep. 2019 Mar 29;9:5380. doi: 10.1038/s41598-019-41851-5 (PMC6441057; doi:10.1038/s41598-019-41851-5)
Supplement: Supplementary file 1 — Supplementary figures and legends clean [file 41598_2019_41851_MOESM1_ESM.docx]

**Impaired hippocampal-cortical coupling but preserved local synchrony during sleep in APP/PS1 mice modeling Alzheimer’s disease**

Zhurakovskaya E^1^, Ishchenko I^1,2^, Gureviciene I^1^, Aliev R^3,4^, Gröhn O^1^, Tanila H^1^*

^1^A. I. Virtanen Institute, University of Eastern Finland, Kuopio Finland
^2^D.I. Ivanovsky Academy of Biology and Biotechnology, Southern Federal University, Rostov-on-Don, Russian Federation
^3^Moscow Institute of Physics and Technology, Moscow, Russian Federation
^4^Institute of Theoretical and Experimental Biophysics, Puschino, Russia

**Supplementary figures**

**Supplementary Figure 1**. **A.** Aimed location of the implanted electrode bundles (sections) and skull screw electrodes (right). mFC = medial frontal cortex, Th-RT = thalamic reticulate nucleus, CA3, CA1 cornu ammonis area 3/1, DG = dentage gyrus, RS = retrosplenial cortex, ref = reference electrodes. This figure is not covered by the CC BY licence. Sections and distance from bregma are taken from (Paxinos and Franklin, 2001) with a permission from Elsevier*. **B**. Histologically verified locations of the electrode tip in transgenic mice (red dots) and **C.** in wild-type mice (blue dots).

Paxinos G, Franklin KBJ. The Mouse Brain in Stereotaxic Coordinates, 2^nd^ ed., 2001. Academic Press, San Diego, CA, USA.

* Reuse of these figures requires permission from Elsevier

**Supplementary Figure 2**. Examples of the extent of amyloid pathology of APP/PS1 mice at the end of the study. A. Mouse with the heaviest amyloid load, B. mouse with the lightest amyloid load. Staining for human specific N-terminal Aβ antibody WO2. Scale bar = 1.0 mm.


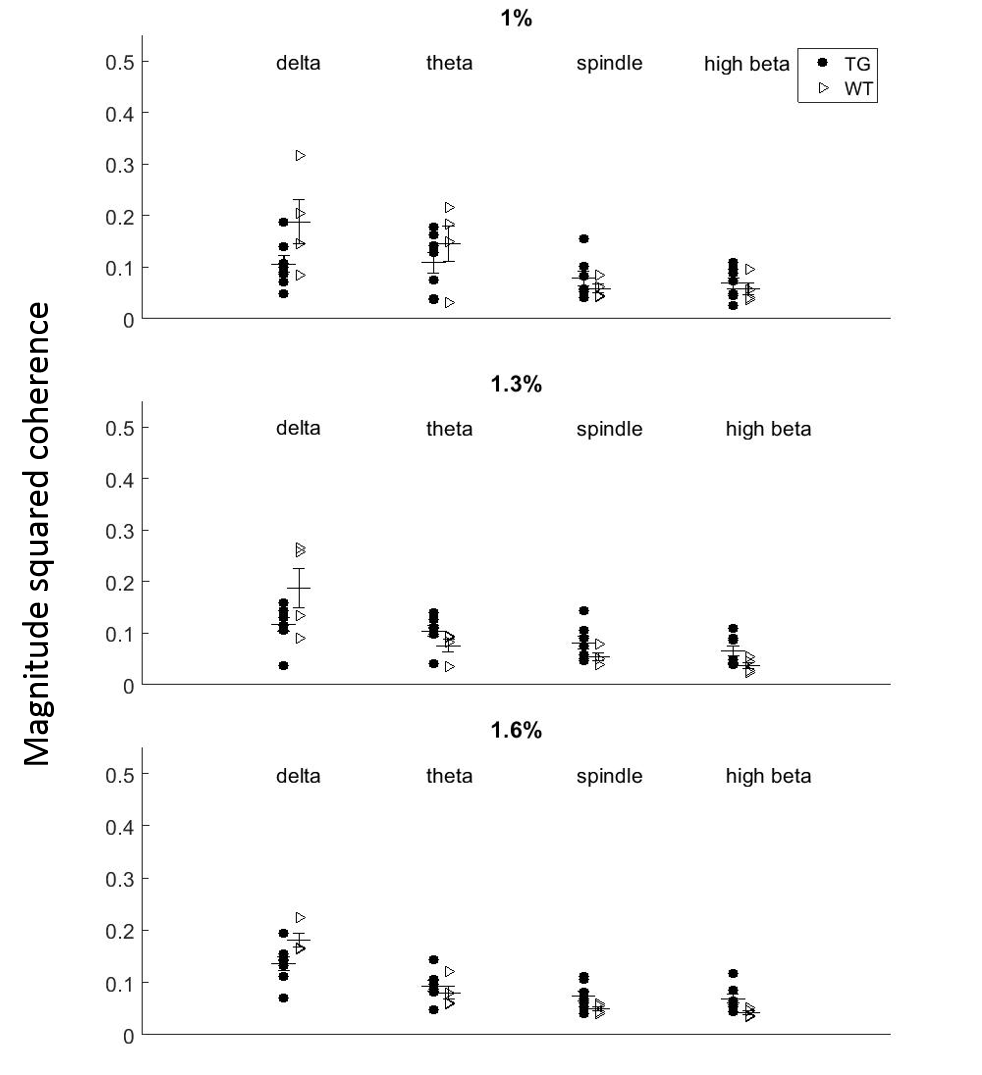


**Supplementary Figure 3**. Connectivity between medial frontal cortex (mFC) and CA1 during isoflurane anesthesia in different isoflurane concentrations (1 – 1.6%) measured with magnitude squared coherence in delta, theta, spindle and high-beta frequency bands. There was a similar trend as during NREM sleep for TG mice to show a lower connectivity but the trend did not reach significance (all p > 0.07, uncorrected). Number of animals used: 7 TG and 4 WT.
